# Cyberaddiction among Tunisian Adolescents: Prevalence and associated factors

**DOI:** 10.1192/j.eurpsy.2026.10856

**Published:** 2026-07-31

**Authors:** Z. Mallek, M. Trigui, M. Baklouti, Y. Mejdoub, E. Mziou, F. Ben Dhaou, J. Jdidi, S. Yaich, M. Kassis

**Affiliations:** 1 Hygiene, Habib Bourguiba University Hospital; 2 Social Medicine, Faculty of medicine; 3 Community Health and Epidemiology, Hedi Chaker University Hospital, Sfax, Tunisia

## Abstract

**Introduction:**

Internet use has become an integral part of adolescents’ daily lives. However, excessive or uncontrolled use can lead to cyberaddiction, which may negatively impact academic performance, social interactions, and mental health.

**Objectives:**

We aimed to estimate the prevalence of cyberaddiction among adolescents in Sfax, Tunisia, and to identify its associated factors.

**Methods:**

A cross-sectional study was conducted among adolescents attending middle and high schools in urban and rural areas of Sfax, Tunisia, in October 2024. An anonymous self-administered questionnaire was distributed to adolescents, ensuring anonymity and confidentiality of responses. Cyberaddiction was measured using the 20-item Internet Addiction Test (IAT) in its Arabic version. Cyberaddiction was defined as an Internet Addiction Test score ≥50.

**Results:**

We included 1,123 adolescents with a sex ratio of 0.64. Among them,65.8% (n=739) were from urban areas, and 19.6% (n=218) had high socioeconomic status. When asked about their internet use,88.9% (n=998) reported using the internet daily, while 7.6% (n=85) used it at least once a week. The prevalence of cyberaddiction among adolescents was 60.4% (n=678), with 28.8% (n=323) exhibiting moderate addiction, and 1.9% (n=21) exhibiting severe internet addiction. The analysis of factors associated with cyberaddiction among adolescents showed that it was significantly more common among those who connected to the internet during class (Odds Ratio (OR) = 4.7; p < 0.001), among adolescents from urban areas (OR = 1.5; p = 0.001), and among high school students (OR = 1.4; p = 0.005). Furthermore, cyberaddiction was more frequent among those who had a high socioeconomic status compared to those who had a low socioeconomic status (OR=1.44; p=0.029).

**Conclusions:**

Our study showed that cyberaddiction is highly prevalent among adolescents in Tunisia, exceeding prevalence estimates from studies conducted in other countries. Therefore, preventive strategies should include school-based educational programs on responsible internet use, parental monitoring and guidance, and targeted interventions for high-risk adolescents to reduce cyberaddiction and its associated consequences.

**Disclosure of Interest:**

None Declared

